# Supplementary material for: Downregulation of Zebrafish Cytosolic Sialidase Neu3.2 Affects Skeletal Muscle Development
Source: Int J Mol Sci. 2023 Sep 1;24(17):13578. doi: 10.3390/ijms241713578 (PMC10487903; doi:10.3390/ijms241713578)
Supplement: Supplementary file 1 [file ijms-24-13578-s001.zip › ijms-2586799-supplementary.pdf]

Daniela Zizioli et al. Downregulation of Zebrafish Cytosolic Sialidase Neu3.2 Affects Skeletal Muscle Development

**Supplementary materials**

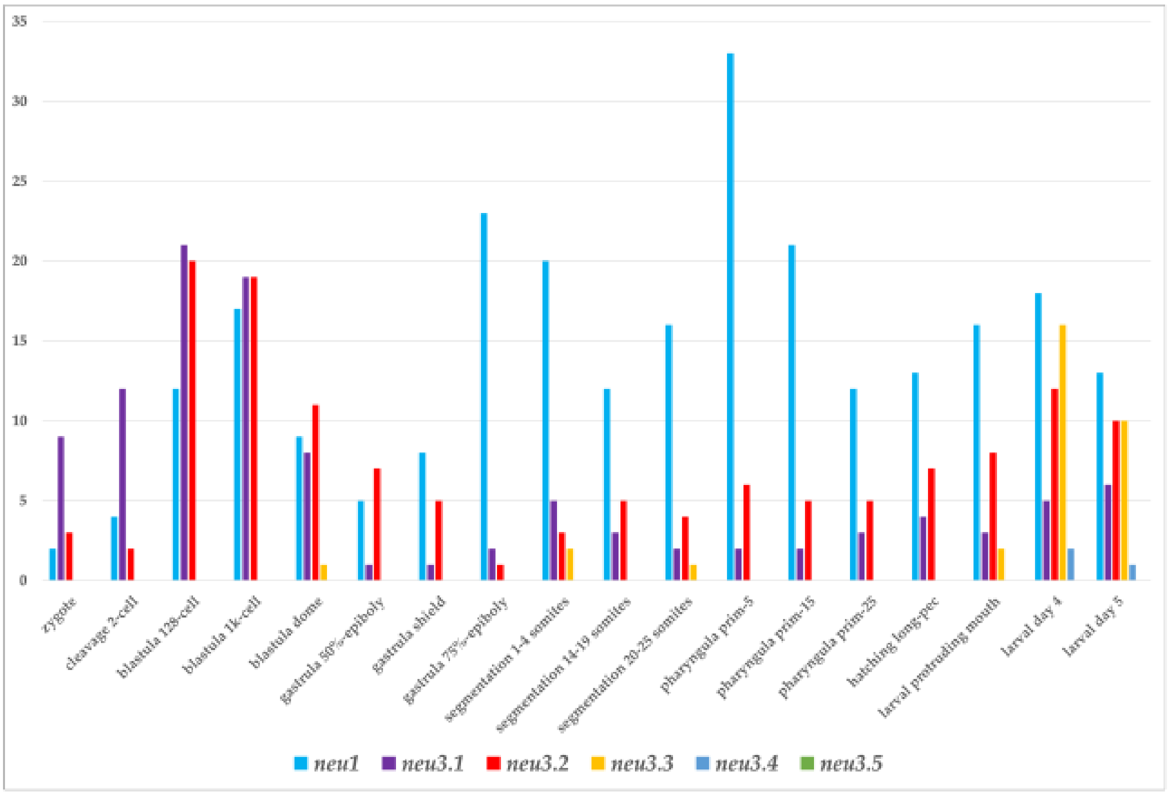


**Figure S1 Expression data for *neu* genes during zebrafish development**.
Expression data for the seven *Danio rerio* sialidase genes obtained from a systematic study performed using RNA-Seq across 18 developmental time points from 1 cell to 5 days post-fertilization [8]. Expression values are expressed in Transcripts Per Kilobase Million) (TPM).

**Expression profile of *neu3.2* during embryonic development**

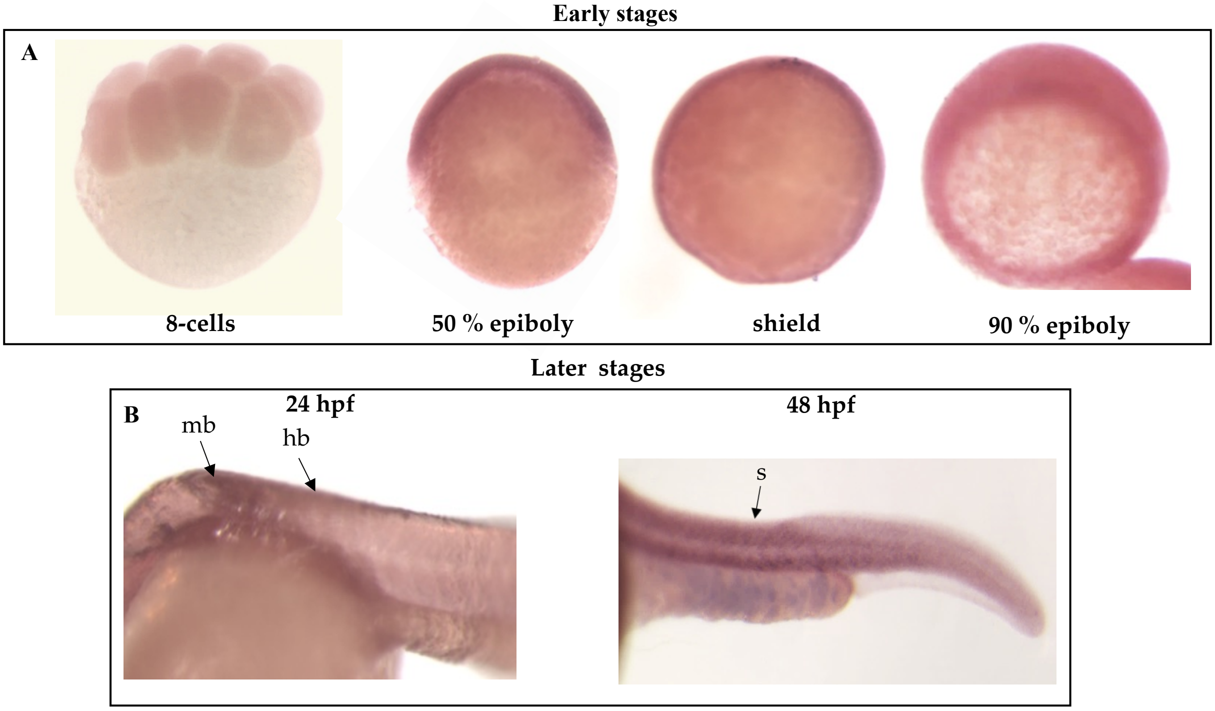


**Figure S2 Spatial expression of *neu3.2* at different embryonic stages**

Stereomicroscope images of ISH analysis on embryos at different developmental stages using an antisense probe against *neu3.2* mRNA. All embryos are lateral views and head pointing to the left. (24 and 48 hpf) with the animal pole up (8 cells, epiboly and shield). A) early stages: 50 % epiboly, shield; 90 % epiboly; B) later stages: 24 and 48 hpf. Data were generated from three biological replicates, pool of 25 embryos for each stage. Abbreviations: **mb**, midbrain; **hb**, hindbrain; **s**, somites

**Dose curve of *neu3.2*-MO**

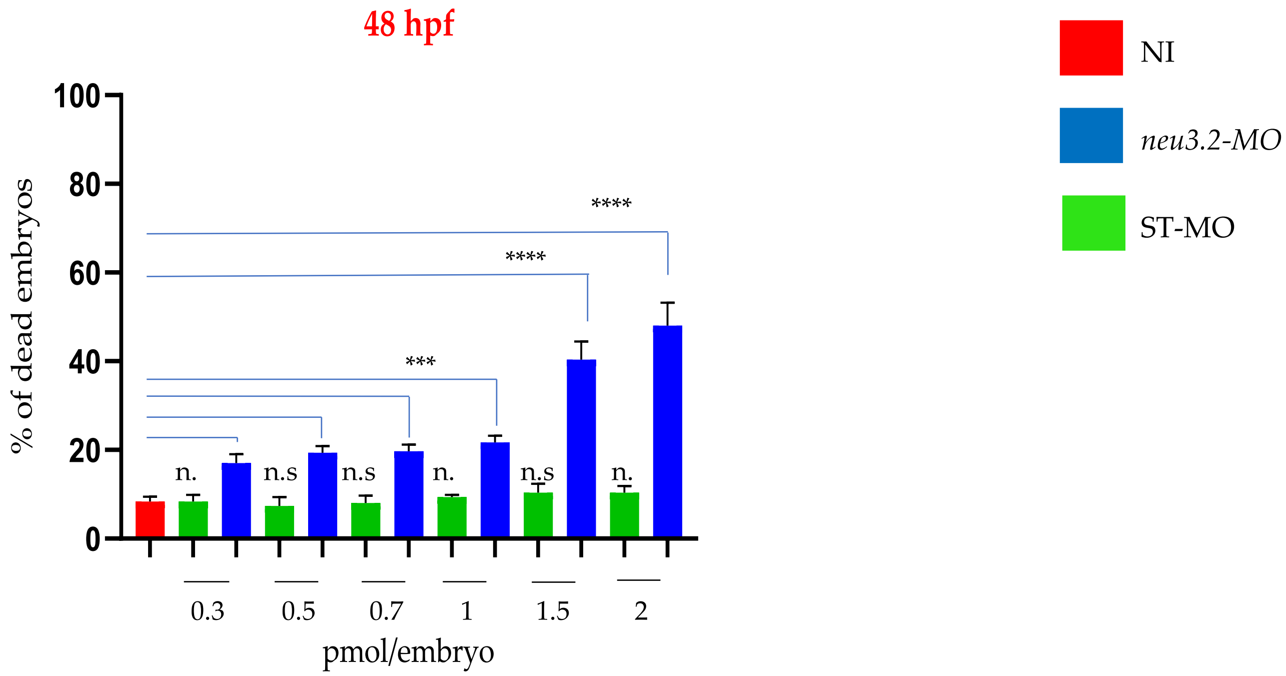


**Figure S3 Dose curve of *neu3.2*-MO 48 hpf**

Lethality rates of zebrafish embryos at 48 hours post fertilization (hpf) after morpholino injection. NI represents the not injected embryos; *neu3.2*-MO represents the embryos injected with different doses of *neu3.2*-MO at one cell stage (0,3; 0,5; 0,7; 1, 1,5 and 2 pmol/embryo); ST-Mo represents the embryos injected at one cell stage with the same doses of ST-MO (Gene Tools, the sequence is reported in math. and meth.) The *X*-axis shows the injected doses expressed in pmol/embryo; the *Y*-axis shows the corresponding lethality expressed in percentages. Results are expressed as mean ± SD of 5 independent experiments, with 180 embryos for each experiment. (*$p < 0.05$ vs. control group; **$p < 0.005$ vs. control group; ***$p < 0.001$ vs. control group; ****$p < 0.0001$ vs. control group; ns: not significative)

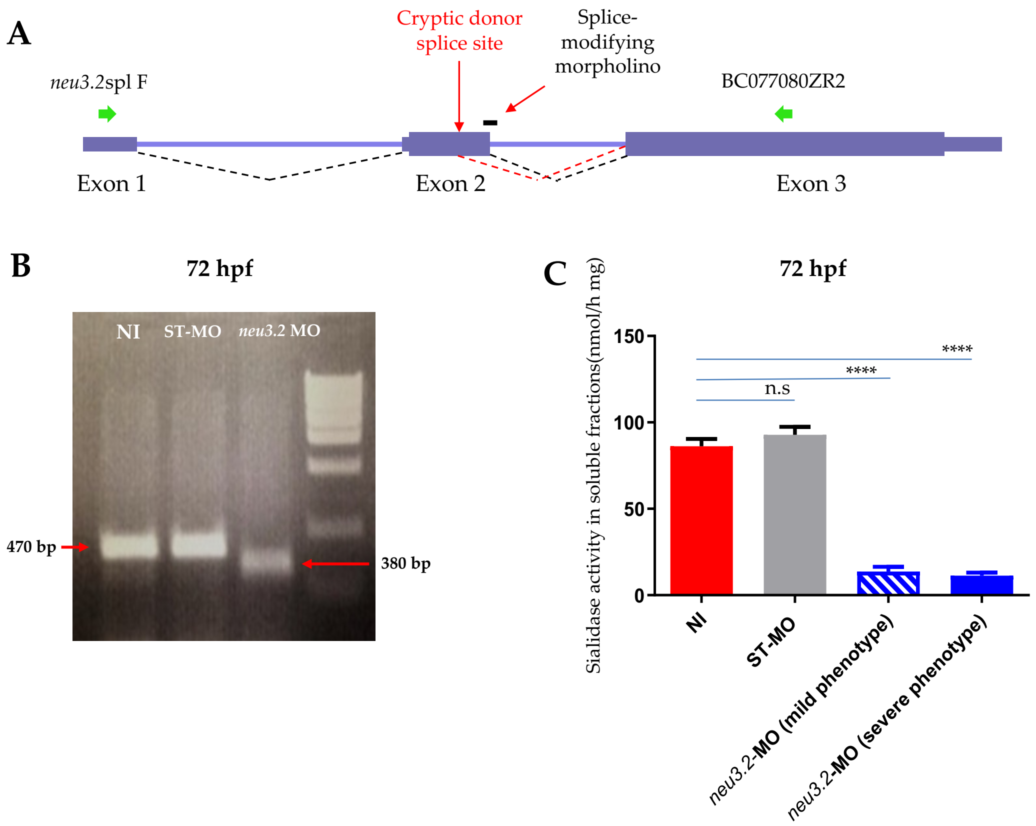


**Figure S4 Efficacy of *neu3.2*-MO and sialidase Neu3.2 activity in embryo extracts**

A) Structure of *neu 3.2* gene with the position of the *neu3.2*-MO and of the oligonucleotide primers used for RT-PCR.

B) RT-PCR analysis of 48 hpf *neu 3.2* morphants shows that *neu3.2*-MO morpholino, targeting intron 2-exon2 junction, interferes with the correct splicing of the primary gene transcript by activating a cryptic donor splice site in exon 2, leading to the generation of a shorter RT-PCR product. NI: not injected embryos; ST-MO: embryos injected with standard morpholino; *neu3.2*-MO: embryos injected with splice morpholino.

C) Sialidase activity in soluble fraction. Assay were performed with up to 10 µg of total proteins. Neu 3.2 activity in soluble fraction was measured using 4-MU-NeuAc as substrate at pH = 5.6. The specific activity was measured in NI embryos, embryos injected with standard morpholino (ST-MO) and injected with neu3.2-MO with severe and mild phenotype. Values are the mean ± SD of three independent experiments (**** $p < 0.0001$ vs not injected embryos; ns = not significative Unpaired, Two-tailed *T*-test) The figure show one representative out of three experiments.

**Dose curve of *Mus musculus Neu2* mRNA**

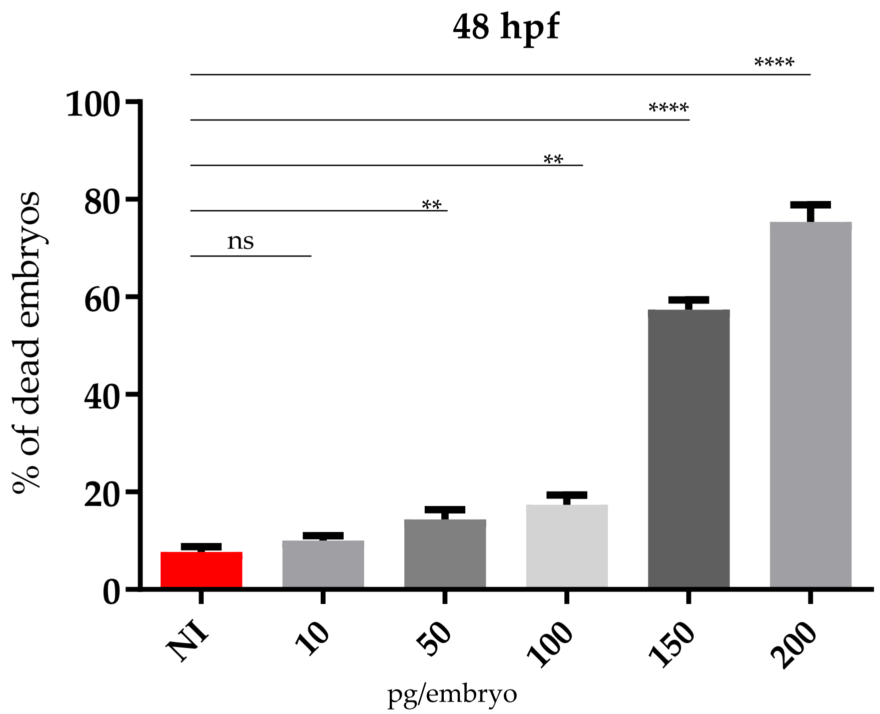


**Figure S5 Dose curve of Neu2 mRNA from *Mus musculus*.**

The percentage of dead embryos after injection of mRNA of *Neu2* from *Mus musculus* was analyzed at 48 hpf. NI represents the not injected embryos. The *X*-axis shows the injected doses expressed in pg/embryo; the *Y*-axis shows the corresponding lethality expressed in percentages. Results are expressed as mean ± SD of 5 independent experiments, with 150 embryos for each experiment. (**p*< 0.05 vs. control group; ***p*< 0.005 vs. control group; ****p*< 0.001 vs. control group; *****p*< 0.0001 vs. control group; ns: not significative)

**Motor behaviour test**

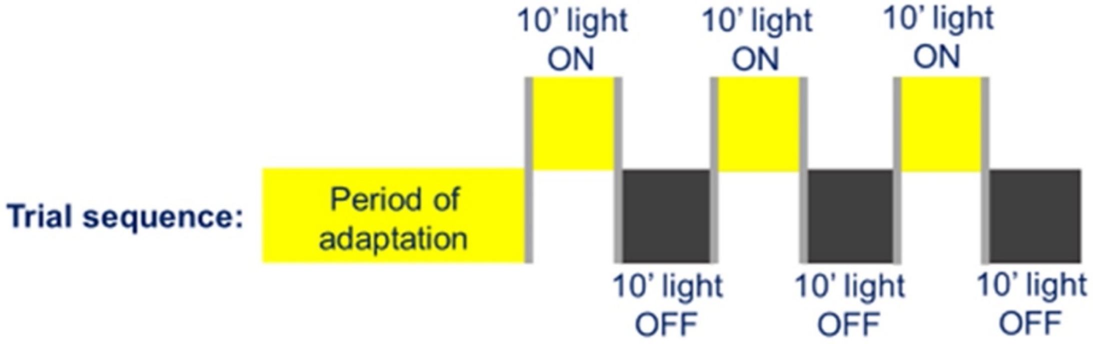


Protocol assessed to track the distance traveled of each zebrafish larvae during the trial.

**Figure S6 Scheme depicting the light-dark locomotor test of larvae at 6 day post fertilization**

The locomotor activity and movement patterns of zebrafish larvae are analyzed by placing them in multi-well plates to record the movements and the travelled distance. In a closed room, after acclimatizing for a while, expose them to varying light and dark. When exposed to changing light and darkness, zebrafish larvae exhibit unique movement patterns, The experimental scheme provides a time (30 minutes) for acclimatation and then 6 cycles of 10 minutes dark and light.

**Table S1:** ***Danio rerio neu3.2*** **NCBI orthologs**

| **Species** | **Common name** | **Amino acids** | **Percentage identity to zebrafish neu3.2** |
|---|---|---|---|
| *Danio rerio* | Zebrafish | 376 | 100 |
| *Danio aesculapii* | | 376 | 96,54 |
| *Sinocyclocheilus rhinocerous* | | 380 | 76,33 |
| *Sinocyclocheilus anshuiensis* | | 381 | 76,06 |
| *Ctenopharyngodon idella* | Grass carp | 469 | 75 |
| *Pimephales promelas* | Fathead minnow | 385 | 71,54 |
| *Myxocyprinus asiaticus* | Chinese sucker | 385 | 69,6 |
| *Xyrauchen texanus* | Razorback sucker | 385 | 69,6 |
| *Triplophysa rosa* | | 388 | 66,49 |
